# Untargeted metabolomics analysis in drug-naïve patients with severe obsessive–compulsive disorder

**DOI:** 10.3389/fnins.2023.1148971

**Published:** 2023-06-02

**Authors:** Zheqin Li, Jian Gao, Liangjun Lin, Zifeng Zheng, Susu Yan, Weidi Wang, Dongdong Shi, Zhen Wang

**Affiliations:** ^1^Shanghai Mental Health Center, Shanghai Jiao Tong University School of Medicine, Shanghai, China; ^2^Shandong Daizhuang Hospital, Jining, Shandong, China; ^3^Shanghai Mental Health Center, School of Biomedical Engineering, Shanghai Jiao Tong University School of Medicine, Shanghai, China; ^4^Shanghai Key Laboratory of Psychotic Disorders, Shanghai Mental Health Center, Shanghai, China; ^5^Brain Science and Technology Research Center, Shanghai Jiao Tong University, Shanghai, China; ^6^Institute of Psychological and Behavioral Science, Shanghai Jiao Tong University, Shanghai, China

**Keywords:** obsessive–compulsive disorder (OCD), metabolomics, untargeted metabolomics analysis, biomarker, WGCNA (weighted gene co- expression network analyses), Unsaturared fatty acid, amino acid, neuroinflammation

## Abstract

**Introduction:**

Obsessive–compulsive disorder (OCD), characterized by the presence of obsessions and/or compulsions, is often difficult to diagnose and treat in routine clinical practice. The candidate circulating biomarkers and primary metabolic pathway alteration of plasma in OCD remain poorly understood.

**Methods:**

We recruited 32 drug-naïve patients with severe OCD and 32 compared healthy controls and applied the untargeted metabolomics approach by ultra-performance liquid chromatography-quadrupole time-of-flight mass spectrometry (UPLC-Q-TOF/MS) to assess their circulating metabolic profiles. Both univariate and multivariate analyses were then utilized to filtrate differential metabolites between patients and healthy controls, and weighted Correlation Network Analysis (WGCNA) was utilized to screen out hub metabolites.

**Results:**

A total of 929 metabolites were identified, including 34 differential metabolites and 51 hub metabolites, with an overlap of 13 metabolites. Notably, the following enrichment analyses underlined the importance of unsaturated fatty acids and tryptophan metabolism alterations in OCD. Metabolites of these pathways in plasma appeared to be promising biomarkers, such as Docosapentaenoic acid and 5-Hydroxytryptophan, which may be biomarkers for OCD identification and prediction of sertraline treatment outcome, respectively.

**Conclusion:**

Our findings revealed alterations in the circulating metabolome and the potential utility of plasma metabolites as promising biomarkers in OCD.

## 1. Introduction

Obsessive–compulsive disorder (OCD) is a common, chronic and severe neuropsychiatric disorder characterized by the presence of obsessions and/or compulsions that lead to various degrees of the subjective experience of anxiety (Veale and Roberts, 2014). About 2–3% of the population suffer from OCD (Fontenelle et al., 2006), and their social relations, quality of life, and occupational functioning were disturbed, costing heavy burdens for their families and society (Yang et al., 2021). Patients with OCD have distinctly heterogeneous obsessive–compulsive symptom dimensions, including contamination, symmetry, checking and unacceptability (Mataix-Cols et al., 2005), which are often obscured by symptoms of anxiety or depression, contributing to the difficulty of OCD diagnosis in routine clinical practice. Whereas up to 40–60% of patients are unable to have a satisfactory outcome through selective serotonin reuptake inhibitors (SSRIs) monotherapy, the proven first-line treatment of OCD (Pallanti and Quercioli, 2006), but may benefit from the combination therapy, cognitive-behavioral therapy or other treatment options. So, it would be helpful if patients who would respond to SSRIs could be identified. Given that, the demand for reliable biomarkers of OCD identification and prediction of sertraline treatment outcome has always been pressing.

Integrating existing neurobiology, genetics and neurochemistry findings, the perturbation of serotonergic (Goddard et al., 2008), dopaminergic (Koo et al., 2010) and glutamatergic (Pittenger et al., 2011) neurotransmitter systems, oxidative imbalance (Maia et al., 2019; Mohammadi et al., 2021) as well as neuroinflammation (Attwells et al., 2017; Gerentes et al., 2019) have been regarded as the underlying neural and pathophysiological underpinnings of OCD (Pauls et al., 2014). Magnetic Resonance Spectroscopy (MRS) studies has revealed glutamatergic dysfunction in cortico-striatal-thalamo-cortical (CSTC) circuit in OCD (Brennan et al., 2013) and SSRIs might act by modulating cortico-striatal glutamatergic activity(Lázaro et al., 2012). But to date, the details of the metabolic pathways and peripheral biomarkers in OCD remain poorly understood. A preceding study applied mendelian randomization design to data of genome-wide association studies (GWAS) to screen blood metabolome among psychiatric disorders but failed to identify any significant biomarker in OCD (Jia et al., 2022). Whilst, a couple of transmitters and their precursors and primary metabolites were probed in blood and Cerebrospinal Fluid (CSF) samples of OCD patients(Benkelfat et al., 1991; Hollander et al., 1992; Swedo et al., 1992; Leckman et al., 1995; Zohar et al., 2000; Chakrabarty et al., 2005; Delorme et al., 2005; Bhattacharyya et al., 2009). Among them, CSF glutamate level (Chakrabarty et al., 2005; Bhattacharyya et al., 2009), plasma homovanillic acid level (the major terminal metabolite of dopamine) (Benkelfat et al., 1991; Hollander et al., 1992) and whole-blood serotonin level (Delorme et al., 2005) were detected to be different in drug-naïve patients compared to healthy controls in some cases. However, these findings were either equivocal or conflicting. To discover biomarkers of OCD, more precise studies will be needed.

High-throughput sequencing technologies have revolutionized medical research. Varieties of omics technologies have been utilized for biomarkers detection and potential pathogenesis interpretation (Hasin et al., 2017). Metabolomics, the newest generation of omics technology, presents the profiles of small molecular compounds in biological samples and reflects directly the underlying biochemical activity and state, as a result of high-throughput characterization of metabolites (Rinschen et al., 2019; Wishart, 2019). The most commonly used metabolomics method is Ultra-performance liquid chromatography-quadrupole time-of-flight mass spectrometry (UPLC-Q-TOF/MS), developed from Liquid Chromatography-Mass Spectrometry (LC–MS), which provides accurate mass information and high mass resolution; helps the analysis of trace compounds in complex multicomponent mixtures like plasma (Alseekh et al., 2021).

Nowadays, metabolomics technologies have been widely applied to discover biomarkers and key pathways in many mental diseases, such as schizophrenia, bipolar disease and depression. However, there has been no study directly and systematically detecting the plasma metabolome in OCD patients yet. In the present study, we aimed to identify the disturbing profile of metabolome in OCD. Consequently, we applied the untargeted metabolomics approach based on UPLC-Q-TOF/MS technology to disclose the primary metabolic pathway alteration in OCD compared with healthy control, and then discovered the candidate biomarker for OCD diagnosis and outcome prediction of sertraline monotherapy.

## 2. Method

**Figure 1:**
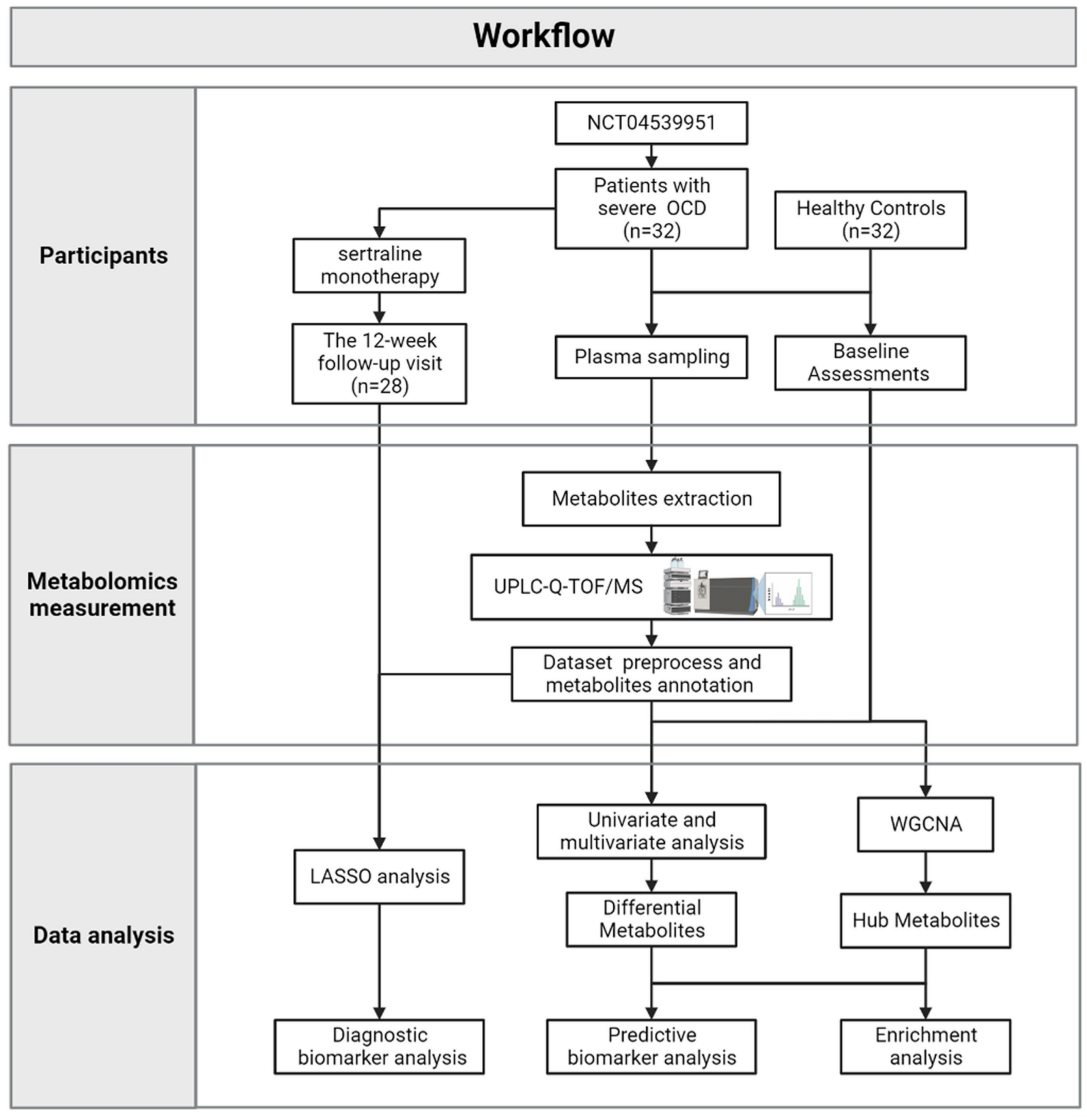
The workflow of the current research.

Figure 1 depicted the integrated workflow of our research. All analyses were conducted by IBM Statistical Product and Service Solutions (SPSS) for Windows, version 26.0 (IBM Corp., Armonk, N.Y., United States), R software, version 4.2.2 and MetaboAnalyst 5.0 Web Server.^1^ The networks were visualized by Cytoscape version 3.9.1.

### 2.1. Participants and procedure

Patients with OCD were recruited at psychiatric clinics of Shanghai Mental Health Center (Shanghai, China) from January 2021 to October 2022 (based on “A Pragmatic Trial of Pharmacotherapy Options Following Unsatisfactory Initial Treatment in OCD” (NCT04539951)). All participants were assessed by experienced psychiatrists who received regular training to ensure consistency. The study was approved by the Institutional Review Board.

Individuals were included in the current study if they: (1) met the Diagnostic and Statistical Manual of Mental Disorder, fifth Edition (DSM-5) criteria for OCD as the primary diagnosis(American Psychological Association, 2013); (2) were OCD patients with a Yale-Brown Obsessive–Compulsive Scale (Y-BOCS) (Goodman et al., 1989) total severity score of ≥24; (3) were 18–65 years old; (4) neither had been exposed to any psychiatric medication nor had received any form of psychotherapy; and (5) had provided written informed consent.

Individuals were excluded if they: (1) met the DSM-5 diagnostic criteria for Schizophrenia Spectrum and Other Psychotic Disorders, or Bipolar and Related Disorders; (2) ever had moderate to severe suicidal ideation or suicide attempts; (3) had psychoactive substance use; (4) had comorbid other medical disorders that may impact on metabolites; (5) were pregnant or lactating females.

Healthy controls, without any DSM-5 diagnosis, were recruited by advertisements. Other inclusion criteria included: (1) between the age of 18 and 65 years; (2) neither had been exposed to any psychiatric medication nor had received any form of psychotherapy; and (3) had provided written informed consent. Individuals were excluded if they: (1) had psychoactive substance abuse; (2) had medical disorders that may impact metabolites; (3) had a family history of mental disorders; or (4) were experiencing pregnancy or lactation.

From January 2021 to October 2022, 58 patients were recruited in the Pragmatic Trial of Obsessive–compulsive Disorder at Shanghai Mental Health Center. Among them, 19 were excluded because their Y-BOCS scores were under 24; 5 were excluded because they received psychiatric medicine before; 2 were excluded because their plasma samples appeared to show signs of hemolysis. As a result, 32 patients with OCD and 32 matched healthy controls (HCs) were included in the present study.

Besides, according to the protocol of “A Pragmatic Trial of Pharmacotherapy Options Following Unsatisfactory Initial Treatment in OCD” (Wang et al., 2022), all included patients with OCD received sertraline (a commonly used SSRIs drug) monotherapy in 12 weeks, initially at 50 mg/d, with a weekly 50 mg/d further increase, to the maximum recommended dosage (200 mg/d) or the maximum tolerated dosage (less than 200 mg/d). Patients would be on their maximum dose by week 4, so allowing an assessment of response at 12 weeks. Till October 2022, there were 28 out of 32 included patients with OCD received 12-week sertraline treatment and finished follow-up visits. Three patients withdrew because of side effects; one patient dropped out because she had an unexpected pregnancy.

As for assessments, the OCD symptom severity of the patient was assessed using the Y-BOCS(Goodman et al., 1989); the Mini International Neuropsychiatric Interview (MINI) was used to screen history of comorbid DSM-5 psychiatric disorders; moreover, the Obsessive–Compulsive Inventory-Revised (OCI-R) (Foa et al., 2002) assesses distress associated with common OCD symptoms, including washing, checking, ordering, obsessing, hoarding, and neutralizing. The Beck Depression Inventory-II (BDI-II) (Beck et al., 1961) and the Beck Anxiety Inventory (BAI) (Beck et al., 1996) were employed to assess the severity of depression and anxiety symptoms, respectively. The details of their clinical characteristic and assessments at baseline were shown in Table 1. The assessments of patients with OCD at the 12-week follow-up visit were shown in Table 2. Responders were defined as patients with the reduction rate of Y-BOCS ≥35%.

**Table 1:** Baseline clinical information of participants.

Group	OCD	HC	*t*.stat	*p*.value
Number, *n*	32	32	/	/
Gender, (male:female)	21:11	21:11	/	/
Age, years	29.84 ± 8.47	30.41 ± 5.65	−0.31	0.756
Education,years	15.5 ± 2.53	14.84 ± 3.65	0.84	0.406
Onset, years	20.91 ± 7.56	/	/	/
Duration, years	7.43 ± 7.85	/	/	/
*Assessment*				
BDI-II, mean ± std.	18.97 ± 9.95	8.69 ± 9.21	4.29	0.000
BAI, mean ± std.	17.69 ± 11.19	3.84 ± 5.25	6.34	0.000
Y-BOCS, mean ± std.	27.75 ± 2.50	/	/	/
Obsession, mean ± std.	14.06 ± 1.32	/	/	/
Compulsion, mean ± std.	13.69 ± 1.69	/	/	/
OCI-R, mean ± std.	27.16 ± 11.57	8.78 ± 6.95	7.70	0.000
Washing, mean ± std.	4.34 ± 3.92	1.44 ± 1.56	3.89	0.000
Checking, mean ± std.	5.84 ± 3.07	1.5 ± 1.50	7.19	0.000
Ordering, mean ± std.	4.13 ± 2.89	1.88 ± 1.68	3.80	0.000
Obsessing, mean ± std.	6.34 ± 2.34	0.91 ± 1.15	11.82	0.000
Hoarding, mean ± std.	2.5 ± 2.65	2 ± 1.80	0.88	0.381
Neutralizing, mean ± std.	4 ± 3.16	1 ± 1.39	4.91	0.000

BDI-II, the Beck Depression Inventory-II; BAI, the Beck Anxiety Inventory; Y-BOCS, Yale-Brown Obsessive–Compulsive Scale; OBSESSION, subscale of obsession in Y-BOCS; COMPULSION, subscale of compulsion in Y-BOCS; OCI-R, Obsessive–Compulsive Inventory-Revised; Washing, subscale of washing symptoms in OCI-R; Checking, subscale of checking symptoms in OCI-R; Ordering, subscale of ordering symptoms in OCI-R; Obsessing, subscale of obsessing thoughts in OCI-R; Hoarding, subscale of hoarding symptoms in OCI-R; Neutralizing, subscale of neutralizing symptoms in OCI-R.

**Table 2:** Clinical information of patients who received sertraline and 12-week follow-up visit.

	Baseline visit	12-week follow-up visit
Number, *n*	28	
Gender, (male: female)	20: 8	
Age, mean ± std. (years)	29.79 ± 8.65	
Education,mean ± std. (years)	15.39 ± 2.60	
Y-BOCS, mean ± std.	27.54 ± 2.12	14.14 ± 6.29
OBSESSION, mean ± std.	14.00 ± 1.25	7.07 ± 3.15
COMPULSION, mean ± std.	13.54 ± 1.45	7.07 ± 3.31
BDI-II, mean ± std.	17.79 ± 9.55	6.04 ± 6.4
BAI, mean ± std.	16.89 ± 11.00	6.86 ± 6.89
Responder, *n* (%)	18 (64%)	

BDI-II, the Beck Depression Inventory-II; BAI, the Beck Anxiety Inventory; Y-BOCS, Yale-Brown Obsessive–Compulsive Scale; OBSESSION, subscale of obsession in Y-BOCS; COMPULSION, subscale of compulsion in Y-BOCS; Responser, patients with a reduction rate of Y-BOCS ≥ 35%.

### 2.2. Plasma samples and metabolomics measurement

All participants were asked to fast for up to 8 hours before blood sampling. About 2 mL venous blood samples were collected from all participants in Ethylenediaminetetraacetic acid (EDTA) anticoagulant polypropylene tubes from an antecubital vein between 9 am and 4 pm. Blood samples were centrifuged at 3,000 rpm at 4°C for 15 min for the separation of blood cells and plasma. The separated plasma then was pipetted into respective vials and stored at −80°C until thawed for analysis.

To extract metabolites, 64 plasma samples (100 μL each) were individually mixed with prechilled methanol (400 μL) by well vortexing. The samples were incubated on ice for 5 min and then were centrifuged at 15,000 rpm, 4°C for 5 min. Then the supernatant was diluted to a final concentration containing 53% methanol by LC–MS grade water. The samples were subsequently transferred to a fresh Eppendorf tube and then were centrifuged at 15000 g, 4°C for 10 min. Finally, the supernatant was injected into the UPLC-Q-TOF/MS system.

UPLC-Q-TOF/MS analyses were performed using a Vanquish UHPLC system (Thermo Fisher, Germany) coupled with an Orbitrap Q Exactive™ HF mass spectrometer (Thermo Fisher, Germany) in Biozeron Co., Ltd. (Shanghai, China). Samples were injected onto a Hypesil Gold column (100 × 2.1 mm, 1.9 μm) using a 17-min linear gradient at a flow rate of 0.2 mL/min. The eluents for the positive polarity mode were eluent A (0.1% FA in Water) and eluent B (Methanol). The eluents for the negative polarity mode were eluent A (5 mM ammonium acetate, pH 9.0) and eluent B (Methanol). The solvent gradient was set as follows: 2% B, 1.5 min; 2–100% B, 12.0 min; 100% B, 14.0 min; 100–2% B, 14.1 min; 2% B, 17 min. Q Exactive™ HF mass spectrometer was operated in positive/negative polarity mode with a spray voltage of 3.2 kV, capillary temperature of 320°C, sheath gas flow rate of 40 arb and aux gas flow rate of 10 arb.

To ensure data quality, pooled quality control (QC) samples were first prepared by mixing all of the plasma samples and were analysed throughout the run every 10 injections. Plasma samples were run alongside QC samples to identify impurities of either the solvents or extraction procedure and for checking carryover contamination from intense analytes.

### 2.3. Dataset annotation and preprocess

The raw data files generated by UPLC-Q-TOF/MS were processed using Compound Discoverer 3.1 (CD3.1, Thermo Fisher) to perform peak alignment, peak picking, and quantitation for each metabolite. Core parameters were set as follows: retention time tolerance, 0.2 min; actual mass tolerance, 5 ppm; signal intensity tolerance, 30%; signal/noise ratio, 3; and minimum intensity, 100,000. After that, peak intensities were normalized to the total spectral intensity, and metabolites with the Coefficient of Variance (CV) less than 30% in QC samples were retained. The normalized data were used to predict the formula based on additive ions, molecular ion peaks and fragment ions. And then peaks were matched with the mzCloud,^2^ mzVault and MassList databases to obtain accurate and relative quantitative results. These metabolites were annotated by the Human Metabolome Database (HMDB, https://hmdb.ca/metabolites).

Afterward, the dataset was normalized by Probabilistic Quotient Normalization (HC group), log transformation and auto-scaling to reach optimal normal distribution (Chen et al., 2017). The principal component analysis (PCA) and heatmaps were first used to observe possible outliers in both data of positive and negative polarity modes; as well as the QC presentation on PCA plots was used for analytical validation.

### 2.4. Data analysis

#### 2.4.1. Metabolic profiling and differential metabolites

Metabolic profiling and differential metabolites between the OCD group and HC group were performed by MetaboAnalyst. After outlier exclusion and normalization, the metabolic profiling was presented by PCA and orthogonal partial least-squares-discriminant analysis (OPLS-DA). Meanwhile, OPLS-DA enables to model separately multivariate data (X-predictors) correlated and uncorrelated to the group labels (Y-responses) and thus the first component of OPLS-DA containing group information is suitable for biomarkers detection. To address the overfitting issue, a 2000-time permutation test of the OPLS-DA model was conducted.

As for differential metabolites, we performed both univariate (t-test and fold change, FC) and multivariate level statistical analysis (OPLS-DA) to screen out. The selection was according to the following criteria: (1) False Discovery Rate (FDR) adjusted *p*-value<0.05; (2) FC (OCD/HCs) > 2.0 or < 0.5; and (3) variable importance in the projection value from the OPLS-DA model (VIP) >1.5.

#### 2.4.2. Weighted gene correlation network analysis

Weighted Correlation Network Analysis (WGCNA) (Langfelder and Horvath, 2008), a systems biology method, has been applied in many high-dimensional data sets, including metabolomics. Briefly, WGCNA constructs a weighted metabolites correlation network and identifies several modules of highly interconnected metabolites. We can relate these modules to sample traits, to filter biologically interested modules and hub metabolites of each interested module. *Hub metabolites* were defined as the metabolites with almost the highest connectivity in the metabolite interaction network, which are more likely to be biologically relevant markers according to prior studies.

In the current study, we conducted a weighted metabolite correlation network and related it with dichotomous grouping variables, Y-BOCS scores and other clinical information accessed at baseline. To be specific, soft thresholding power β was estimated based on the criterion of approximate scale-free topology (*R*^2^ fit >0.9), aiming to calculate the adjacency matrix. To minimize the effects of noise and spurious associations, adjacency values were then transformed into a signed topological overlap matrix (TOM). We used the dissimilarity matrix (1-TOM) to produce the hierarchical clustering trees (dendrogram) of metabolites, and module identification was based on the identification of individual brunches by the Dynamic Tree Cut. Each module’s expression profile was summarized into a module eigengene (ME) using the matched module’s first principal component. After constructing the metabolite correlation network and identifying modules, we selected biologically interested modules from module-trait relationship plots. Hub metabolites were then filtrated according to Module Membership (MM) which measured how correlated each metabolite was to the ME of the related module, and Gene Significances (GS) which was defined as the correlation of a metabolite profile with grouping variables in this case. Metabolites in interesting modules with MM > 0.8 or < −0.8 and GS >0.2 or < −0.2 were considered as hub metabolites.

#### 2.4.3. Pathway analysis and biomarker analysis

After filtering differential and hub metabolites, we performed enrichment analysis on the basis of the KEGG (Kyoto Encyclopedia of Genes and Genomes, https://www.genome.jp/kegg/) database (Kanehisa and Goto, 2000).

Potential biomarkers for diagnosis were selected from metabolites that were both differential and hub metabolites. To avoid multicollinearity, we used stepwise logistic regression to filtrate and remain solid significant predictor variables. A nomogram was then established by combining screened-out plasma metabolites based on the logistic regression model. The multivariate receiver operating characteristic (ROC) curve and the area under the curve (AUC) was also calculated to evaluate the discriminative ability of the model. Additionally, the Hosmer–Lemeshow test was performed to further evaluate the consistency between the predicted efficacy and the actual response rate (*p* > 0.05 was considered a good consistency).

To filtrate potential biomarkers of sertraline monotherapy, the dataset of 28 patients with OCD who had finished 12-week follow-up visits with all detected metabolites was enrolled for the least absolute shrinkage and selection operator (LASSO) regression. LASSO is a penalization method that shrinks all regression coefficients and sets the coefficients of many irrelevant features that have no discriminatory power between the classes exactly to zero. It is suitable for high-dimensional data with large features but small samples and also avoids overfitting. To provide a robust generalized performance of a model that best fitted our data, 10-fold cross-validation with a minimum criterion was applied, with these folds being randomly picked. The minimum lambda value was then defined as a cut-off point to minimize the mean cross-validated error and was utilized to filtrate metabolites.

Subsequently, the selected metabolites were used to perform an outcome prediction nomogram based on the logistic regression model. Similar to the diagnostic nomogram mentioned before, we conducted a ROC curve to evaluate the predictive discriminative performance of the model and the Hosmer–Lemeshow test to describe its consistency.

## 3. Results

### 3.1. Metabolic profiling and differential metabolites

Through the untargeted UPLC-Q-TOF/MS metabolomics analysis, 430 metabolite features in positive polarity mode and 499 in negative polarity mode were extracted. Integrating each sample’s performance in the PCA scores plots and heatmaps (Supplementary Figures S1A–D), 3 samples (X1177, X1202 in the HC group and X89 in the OCD group) were excluded from the following analysis. Besides, the PCA scores plots revealed a close clustering of all QC samples, indicating that the analytical system performance was of good quality.

**Figure 2:**
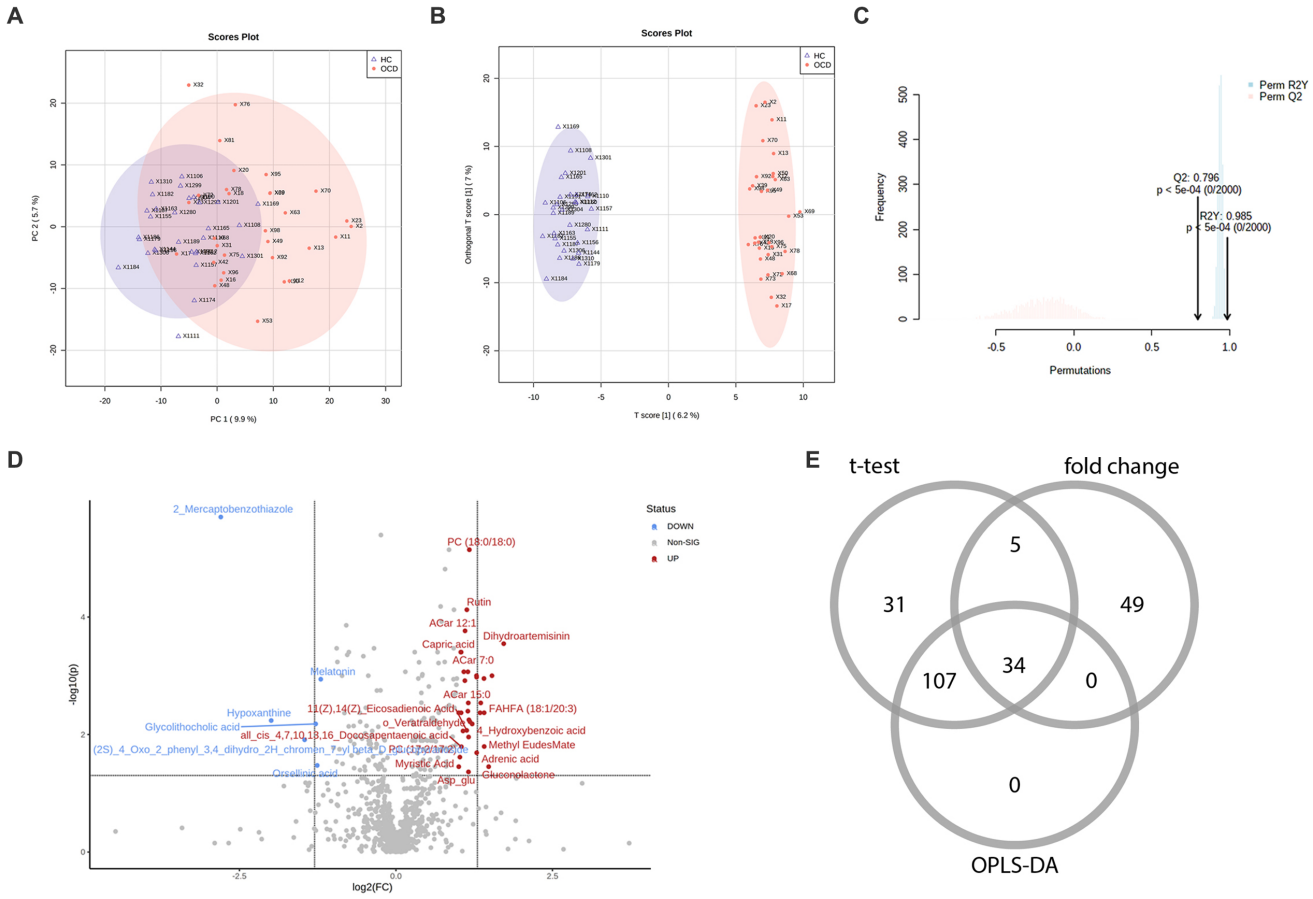
The metabolic profiling between the OCD and HC group and the selection of differential metabolites. **(A)** The scores plot of the principal component analysis between the OCD group and the HC group. **(B)** The scores plot of the orthogonal partial least-squares-discriminant analysis between the OCD and HC group. **(C)** The result of the 2000-time permutation test in the orthogonal partial least-squares-discriminant analysis. **(D)** The volcano plot. **(E)** The Venn diagram of the selection of differential metabolites.

In total, among 929 detected metabolites, there were 475 metabolites showed higher levels in the OCD group and 454 counterparts in the HC group. Metabolic profiling comparing the OCD group with the HC group was first presented in the PCA scores plot (Figure 2A). We can observe an overlap between these two groups, and the HC group seemed to have a more concentrated cluster than the OCD group. A clearer discrimination of metabolic profiling between the OCD and HC groups was presented in Figure 2B, suggesting a better separation with OPLS-DA. The result of the permutation test showed that the classification of the OCD group and HC group was significantly better than any other random classification in two arbitrary groups (Figure 2C; Q2 = 0.796, *p* < 5e-04; R2Y = 0.985, *p* < 5e-04).

According to the univariate and multivariate analysis, 177 metabolites were significantly altered after FDR correction; 88 metabolites met the criterion of FC >2/<0.5. The volcano plot was shown in Figure 2D. There were 141 metabolites detected from the OPLS-DA model. As illustrated in the Venn diagram (Figure 2E), we identified 34 differential metabolites listed in Table 3.

**Table 3:** List of differential metabolites.

Metabolites	HMDB_ID	FC	*t*.stat	*p*.value	FDR	VIP
Uridine 5’ Diphospho N-Acetylgalactosamine	HMDB0000304	2.325	−3.648	0.000561	0.006587	1.725
Rutin	HMDB0003249	2.193	−5.576	6.46E-07	7.51E-05	2.317
Pregnenolone	HMDB0000253	2.439	−4.459	3.76E-05	0.000997	1.973
PC (21:2/20:4)	/	2.896	−4.474	3.56E-05	0.000997	2.079
PC (18:0/18:0)	/	2.255	−6.396	2.82E-08	7.15E-06	2.534
PC (17:0/18:1)	/	2.003	−3.848	0.000295	0.004255	1.893
Palmitic acid	HMDB0000220	2.270	−3.682	0.000503	0.00607	1.781
o-Veratraldehyde	/	2.102	−3.522	0.000833	0.008694	1.640
Methyl EudesMate	/	2.657	−3.218	0.002102	0.016002	1.540
Melatonin	HMDB0001389	0.435	4.367	5.16E-05	0.001144	2.024
Hypoxanthine	HMDB0000157	0.251	3.715	0.000454	0.005777	1.788
Glycolithocholic acid	HMDB0000698	0.411	3.645	0.000567	0.006587	1.787
FAHFA (18:1/20:3)	/	2.558	−4.025	0.000164	0.002898	1.974
Elaidic acid	HMDB0000573	2.655	−3.842	0.000301	0.004255	1.868
Docosapentaenoic acid	HMDB0246621	2.539	−3.863	0.000281	0.004255	1.846
Dihydroartemisinin	HMDB0242686	3.297	−5.108	3.67E-06	0.000284	2.249
Decanoylcarnitine	HMDB0000651	2.218	−4.556	2.67E-05	0.000855	2.152
Capric acid	HMDB0000511	2.049	−4.877	8.51E-06	0.000395	2.234
all-cis-4,7,10,13,16-Docosapentaenoic acid	/	2.063	−3.211	0.002141	0.016169	1.593
Adrenic acid	HMDB0002226	2.444	−3.097	0.002988	0.020413	1.557
ACar 7:0	/	2.058	−4.921	7.26E-06	0.000395	2.202
ACar 15:0	/	2.228	−4.024	0.000165	0.002898	1.918
ACar 13:0	/	2.120	−4.569	2.55E-05	0.000855	2.081
ACar 12:1	/	2.149	−5.268	2.04E-06	0.000172	2.381
ACar 11:0	/	2.145	−4.343	5.60E-05	0.00121	2.033
8Z,11Z,14Z-Eicosatrienoic acid	HMDB0002925	2.243	−3.732	0.00043	0.005623	1.797
4-Hydroxybenzoic acid	HMDB0000500	2.236	−3.429	0.00111	0.011022	1.639
3-Hydroxybutyric acid	HMDB0000011	2.057	−3.852	0.000291	0.004255	1.812
2-Mercaptobenzothiazole	HMDB0030524	0.144	7.061	2.13E-09	1.98E-06	2.736
13,14-dihydro 15-keto Prostaglandin A2	HMDB0001244	2.217	−3.889	0.000258	0.004002	1.790
11(Z),14(Z)-Eicosadienoic Acid	HMDB0005060	2.189	−3.537	0.000796	0.0085	1.742
11(E)-Eicosenoic Acid	/	2.651	−4.403	4.55E-05	0.001113	2.067
(R)-3-Hydroxy myristic acid	/	2.442	−4.434	4.08E-05	0.001054	2.029
(2S) 4-oxo-2-phenyl-3,4-dihydro-2H-chromen-7-yl beta-D-glucopyranoside	/	0.364	3.368	0.001339	0.012289	1.751

HMDB_ID, The Human Metabolome Database accession number; FC, fold change; FDR, The False Discovery Rate-adjusted *p*-value, VIP, variable importance in the projection value.

### 3.2. Weighted metabolite correction network and hub metabolites in all participants

**Figure 3:**
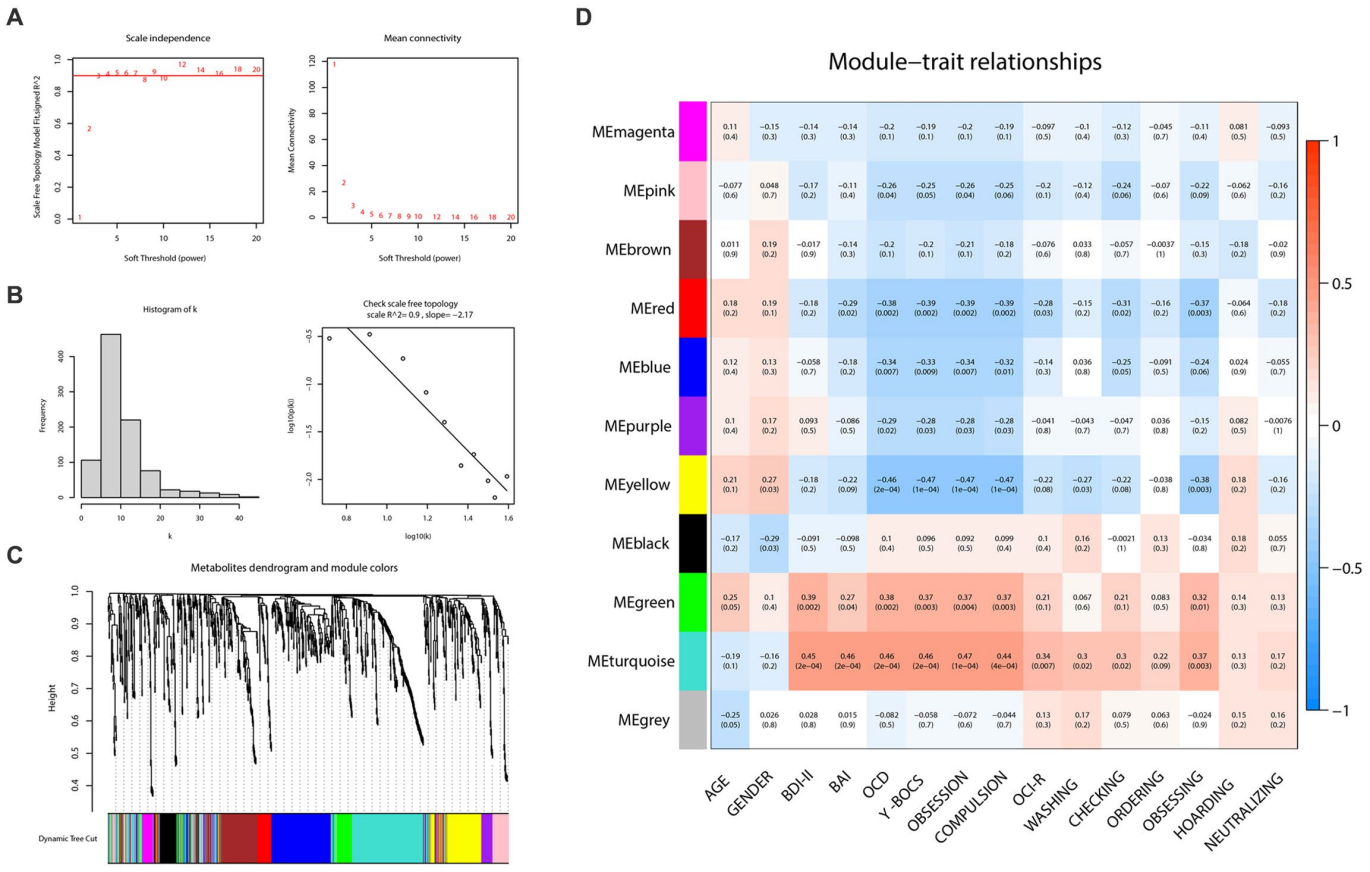
Weighted correlation network analysis and the selection of hub metabolites. **(A)** Scale-free fitting index analysis and mean connectivity of soft threshold power from 1 to 20. **(B)** Checking the scale free topology (power = 3). **(C)** Clustering dendrograms. **(D)** Correlation heatmap between module eigengenes and clinical traits.

To construct the weighted metabolite correction network, power 3, which was the lowest power for which the scale-free topology fit index reaches 0.90, was chosen (Figures 3A,B). Subsequently, there were 11 distinct modules clustered and labeled with colors (Figure 3C) in the weighted metabolite correction network. The grey module was reserved for unassigned metabolites.

As the plot of the module-trait relationship (Figure 3D) showed, grouping variables (namely “OCD” in the plot) and Y-BOCS scores had the highest correction with turquoise (cor = 0.46, *p* = 2e-04; cor = 0.46, *p* = 2e-04, respectively), yellow (cor = −0.46, *p* = 2e-04; cor = −0.47, *p* = 1e-04, respectively), red (cor = −0.38, *p* = 0.002; cor = −0.39, *p* = 0.002, respectively), green (cor = 0.38, *p* = 0.002; cor = 0.37, *p* = 0.003, respectively) and blue modules (cor = −0.34, *p* = 0.007; cor = −0.33, *p* = 0.009, respectively). With regard to OCI-R, it turned out to be similar but slightly different. The total scores of OCI-R saw significant associations with turquoise (cor = 0.34, *p* = 0.007) and red modules (cor = −0.28, *p* = 0.02) rather than the other three mentioned modules. The results in each subscale of OCI-R also differed. Subscales of ordering, hoarding and neutralizing were uncorrected with any module, while the subscale of obsessing was relevant with red (cor = −0.37, *p* = 0.003), yellow (cor = −0.38, *p* = 0.003) and turquoise (cor = 0.37, *p* = 0.003) modules. In addition, the turquoise module was closely related to the BDI-II (cor = 0.45, *p* = 2e-04) and BAI (cor = 0.46, *p* = 2e-04) scores; the green module also represented a prominent positive correlation with BDI-II scores (cor = 0.39, *p* = 0.002).

**Figure 4:**
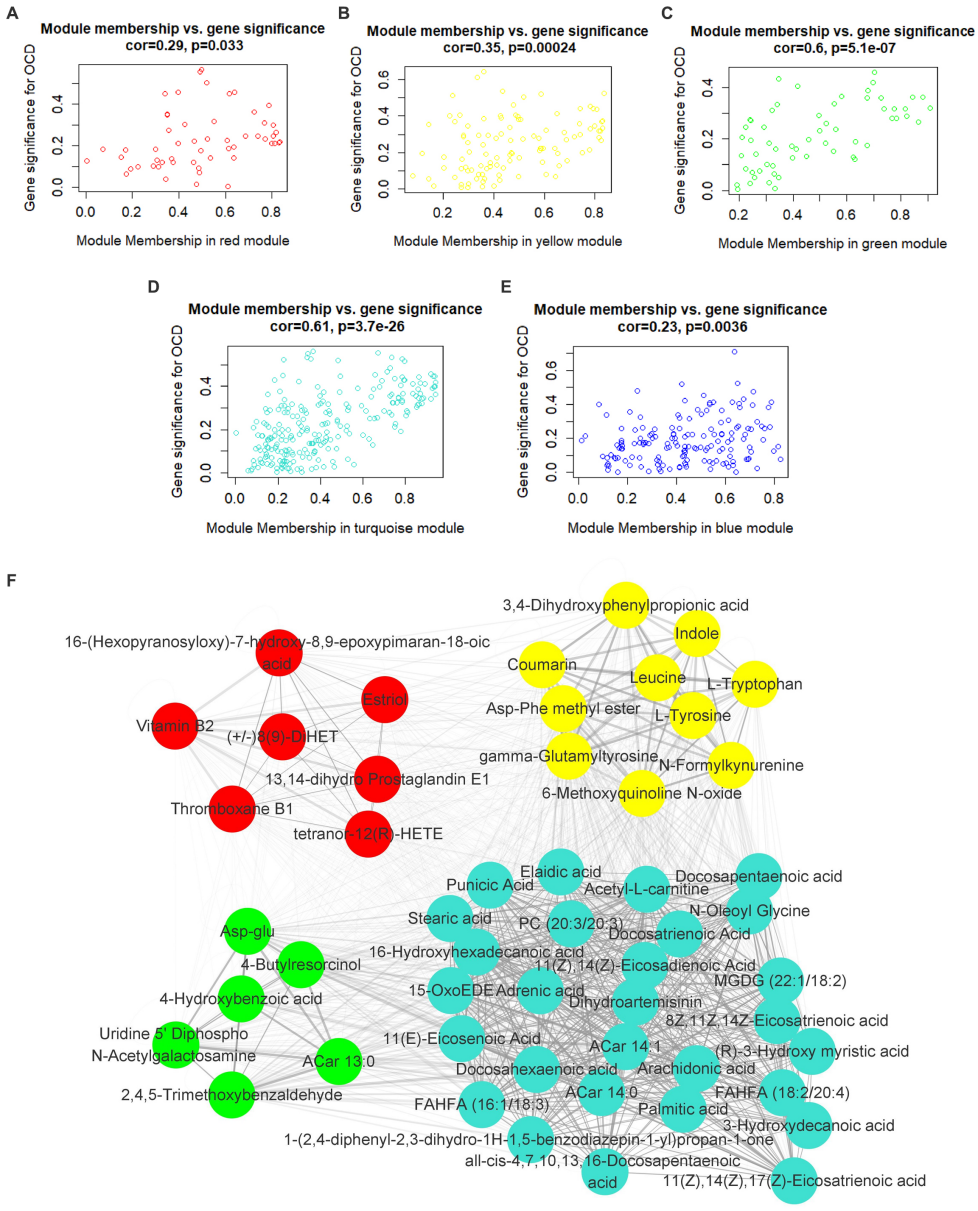
Hub metabolites. Correlation between module membership and gene significance of all metabolites in **(A)** red, **(B)** yellow, **(C)** green, **(D)** turquoise and **(E)** blue module. **(F)** correlation network of 51 selected hub metabolites.

In the light of this, red, blue, yellow, green and turquoise modules were considered as interested modules. Figures 4A–E presented the correlation between MM and GS of all metabolites in each selected module. 51 hub metabolites were filtrated, on the basis of the standards mentioned above in the method, listed in Table 4. None was screened out in the blue module. The correlation network of these hub metabolites was in Figure 4F.

**Table 4:** List of hub metabolites.

Metabolites	HMDB_ID	MM	GS	FC	*t*-stat	*p*-value	FDR	VIP
Red module								
Estriol	HMDB0000153	0.811	−0.210	0.574	2.292	0.025507	0.094721	1.069
tetranor-12(R)-HETE	/	0.800	−0.212	0.473	2.338	0.022811	0.087207	1.107
13,14-dihydro Prostaglandin E1	HMDB0002689	0.832	−0.214	0.378	2.451	0.017245	0.074514	1.171
(+/−)8(9)-DiHET	HMDB0002311	0.832	−0.218	0.368	2.517	0.014569	0.066115	1.188
Thromboxane B1	/	0.806	−0.246	0.527	2.238	0.029013	0.10327	1.052
16-(Hexopyranosyloxy)-7-hydroxy-8,9-epoxypimaran-18-oic acid	/	0.819	−0.263	0.520	2.011	0.048879	0.15604	0.921
Vitamin B2	HMDB0000244	0.806	−0.300	0.779	2.339	0.022747	0.087207	1.075
Yellow module								
L-Tryptophan	HMDB0000929	0.827	−0.266	0.972	0.785	0.43583	0.61815	0.396
Indole	HMDB0000738	0.815	−0.284	0.960	1.065	0.29125	0.48752	0.568
gamma-Glutamyltyrosine	HMDB0011741	0.818	−0.317	0.881	2.072	0.042603	0.14237	1.012
Asp-Phe methyl ester	/	0.822	−0.318	0.918	1.712	0.092167	0.24256	0.881
Leucine	HMDB0000687	0.832	−0.333	0.852	2.285	0.02595	0.094913	1.134
Coumarin	HMDB0001218	0.801	−0.344	0.855	2.461	0.016812	0.073327	1.206
3,4-Dihydroxyphenylpropionic acid	HMDB0000423	0.827	−0.371	0.843	2.896	0.005296	0.031948	1.428
L-Tyrosine	HMDB0000158	0.830	−0.372	0.839	2.903	0.005189	0.031505	1.436
N-Formylkynurenine	HMDB0060485	0.808	−0.439	0.854	2.838	0.006219	0.035247	1.406
6-Methoxyquinoline N-oxide	/	0.836	−0.525	0.883	3.355	0.001391	0.012549	1.593
Green module								
Uridine 5’ Diphospho N-Acetylgalactosamine^*^	HMDB0000304	0.880	0.362	2.325	−3.648	0.00056	0.00659	1.725
ACar 13:0^*^	/	0.844	0.362	2.120	−4.569	2.6E-05	0.00085	2.081
4-Hydroxybenzoic acid^*^	HMDB0000500	0.907	0.320	2.236	−3.429	0.00111	0.01102	1.639
2,4,5-Trimethoxybenzaldehyde	HMDB0029648	0.817	0.318	1.873	−3.223	0.002069	0.015886	1.555
4-Butylresorcinol	/	0.821	0.288	2.236	−3.429	0.00111	0.011022	1.639
Asp-glu	HMDB0028752	0.866	0.267	2.233	−2.738	0.008152	0.043277	1.372
Turpuoise module								
Dihydroartemisinin^*^	HMDB0242686	0.919	0.457	3.297	−5.108	3.7E-06	0.00028	2.249
3-Hydroxydecanoic acid	HMDB0010725	0.938	0.447	1.962	−4.396	4.67E-05	0.001113	2.000
(R)-3-Hydroxy myristic acid^*^	/	0.931	0.445	2.442	−4.434	4.1E-05	0.00105	2.029
Docosahexaenoic acid	HMDB0002183	0.863	0.441	1.897	−4.132	0.000115	0.002137	1.902
Docosapentaenoic acid^*^	HMDB0246621	0.940	0.419	2.539	−3.863	0.00028	0.00425	1.846
Stearic acid	HMDB0000827	0.870	0.415	1.952	−4.289	6.75E-05	0.001426	1.997
ACar 14:1	/	0.834	0.413	1.941	−4.170	0.000101	0.001956	2.041
N-Oleoyl Glycine	HMDB0241916	0.892	0.410	1.892	−4.366	5.17E-05	0.001144	2.070
11(Z),14(Z)-Eicosadienoic Acid^*^	HMDB0005060	0.937	0.401	2.189	−3.537	0.0008	0.0085	1.742
8Z,11Z,14Z-Eicosatrienoic acid^*^	HMDB0002925	0.937	0.397	2.243	−3.732	0.00043	0.00562	1.797
Palmitic acid^*^	HMDB0000220	0.934	0.396	2.270	−3.682	0.0005	0.00607	1.781
Elaidic acid^*^	HMDB0000573	0.928	0.391	2.655	−3.842	0.0003	0.00425	1.868
11(E)-Eicosenoic Acid^*^	/	0.858	0.388	2.651	−4.403	4.6E-05	0.00111	2.067
11(Z),14(Z),17(Z)-Eicosatrienoic acid	/	0.870	0.365	1.479	−3.427	0.001118	0.011022	1.700
ACar 14:0	/	0.835	0.364	1.508	−3.673	0.000518	0.006167	1.774
Adrenic acid^*^	HMDB0002226	0.939	0.364	2.444	−3.097	0.00299	0.02041	1.557
Acetyl-L-carnitine	HMDB0240773	0.850	0.354	1.509	−3.768	0.000382	0.005216	1.755
all-cis-4,7,10,13,16-Docosapentaenoic acid^*^	/	0.864	0.346	2.063	−3.211	0.00214	0.01617	1.593
1-(2,4-diphenyl-2,3-dihydro-1H-1,5-benzodiazepin-1-yl)propan-1-one	/	0.883	0.343	1.785	−3.142	0.002621	0.018304	1.575
Arachidonic acid	HMDB0001043	0.921	0.343	1.593	−3.013	0.003807	0.024864	1.493
PC (20:3/20:3)	/	0.877	0.340	1.554	−3.309	0.001601	0.01305	1.706
FAHFA (18:2/20:4)	/	0.876	0.322	1.540	−2.833	0.006298	0.035247	1.511
MGDG (22:1/18:2)	/	0.861	0.316	1.624	−2.290	0.025592	0.094721	1.276
Docosatrienoic Acid	HMDB0002823	0.854	0.314	1.462	−2.984	0.004136	0.026684	1.482
FAHFA (16:1/18:3)	/	0.851	0.302	2.165	−2.272	0.026765	0.097128	1.115
Punicic Acid	HMDB0030963	0.806	0.285	1.457	−2.659	0.010068	0.051673	1.425
15-OxoEDE	/	0.810	0.279	1.311	−2.925	0.004879	0.030017	1.522
16-Hydroxyhexadecanoic acid	HMDB0006294	0.804	0.265	1.201	−3.334	0.001482	0.012671	1.651

HMDB_ID, The Human Metabolome Database accession number; MM, Module Membership; GS, Gene Significances; FC, fold change; FDR, The False Discovery Rate-adjusted *p*-value, VIP, variable importance in the projection value.

^*^Differential metabolites.

Interestingly, as Figure 4D illustrated, most of the hub metabolites in the turquoise module were fatty acids, which included polyunsaturated fatty acids (PUFAs) such as Arachidonic acid, Docosahexaenoic acid (DHA), Docosapentaenoic acid (DPA), all-cis-4,7,10,13,16-Docosapentaenoic acid, 8Z,11Z,14Z-Eicosatrienoic acid, 11(Z),14(Z)-Eicosadienoic Acid, 11(Z),14(Z),17(Z)-Eicosatrienoic acid, Punicic Acid, Adrenic acid and Docosatrienoic Acid; saturated fatty acids such as Palmitic acid, Stearic acid and Capric acid. In red module (Figure 4A), several eicosanoids, such as tetranor-12(R)-HETE, Thromboxane B1, 13,14-dihydro Prostaglandin E1 and (+/−)8(9)-DiHET were screened as hub metabolites. In the yellow module (Figure 4B), there were several amino acids, such as L-Tryptophan (Trp), L-Tyrosine (Tyr) and Leucine, and related precursors or primary metabolites.

### 3.3. Enrichment analyses of differential and hub metabolites

**Figure 5:**
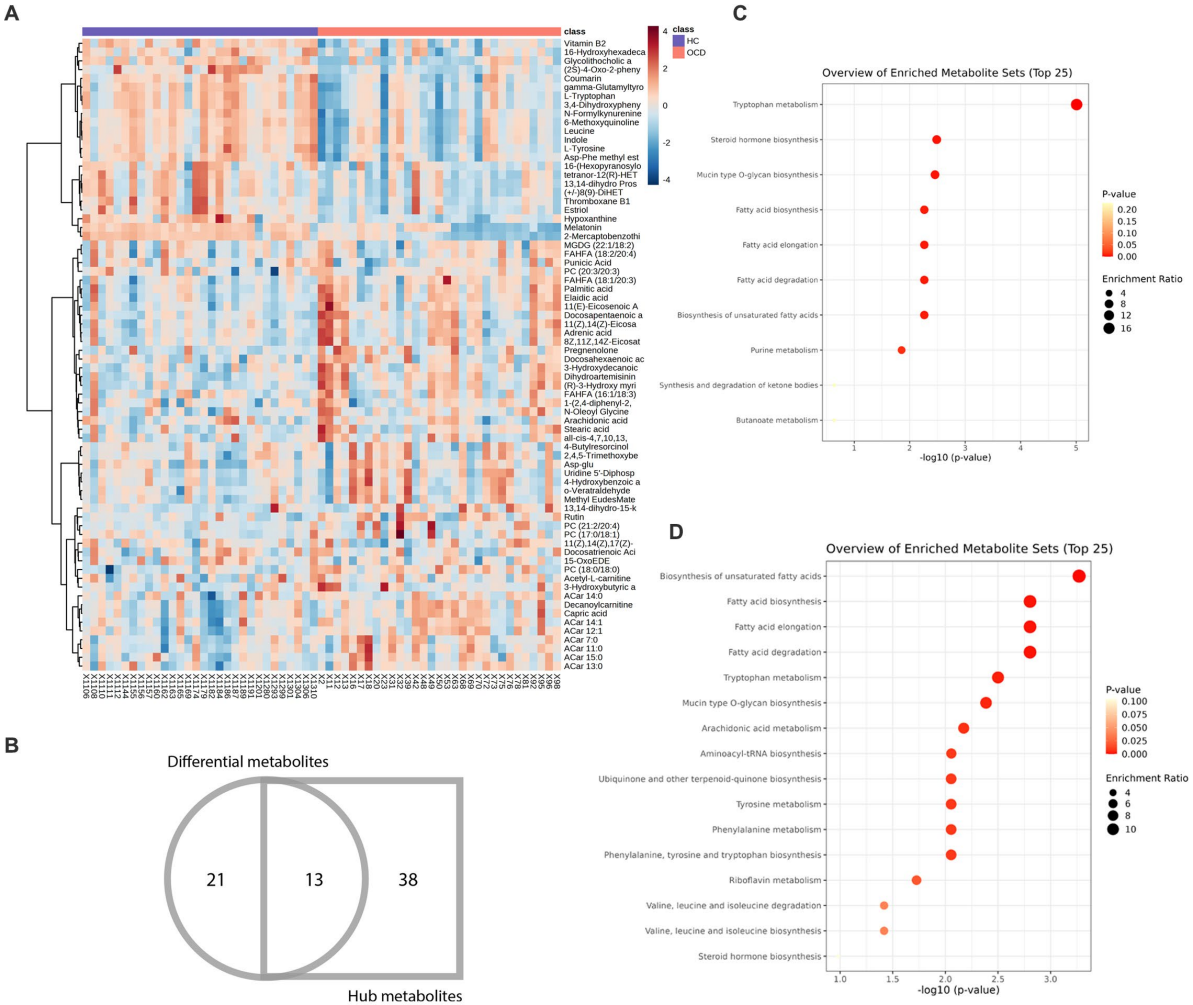
Heatmap and enrichment analyses of differential and hub metabolites. **(A)** Heatmap of differential and hub metabolites. **(B)** The Venn diagram of differential and hub metabolites. **(C)** Enrichment analyses of differential metabolites. **(D)** Enrichment analyses of hub metabolites.

72 metabolites in total were selected as interested metabolites. The heatmap was shown in Figure 5A and the Venn diagrams were illustrated in Figure 5B. While only 19 differential metabolites and 32 hub metabolites were annotated by HMDB (Tables 3, 4) and were included in the following enrichment analysis (Figures 5C,D). As Table 5 listed, in both pathway analysis of differential metabolites and hub metabolites, biosynthesis of unsaturated fatty acids and tryptophan metabolism were detected.

**Table 5:** Enrichment analyses of differential and hub metabolites.

Metabolite set	Total Compound	Hits	Hit metabolites	Statistic *Q*	Expected *Q*	Raw *p*	Holm *p*	FDR
Differential metabolites								
Tryptophan metabolism	41	1	Melatonin	27.224	1.587	0.000	0.000	0.000
Steroid hormone biosynthesis	85	1	Pregnenolone	13.127	1.587	0.003	0.029	0.008
Mucin type O-glycan biosynthesis	10	1	Uridine diphosphate-N-acetylgalactosamine;	12.947	1.587	0.003	0.029	0.008
Fatty acid biosynthesis	47	2	Palmitic acid; Capric acid	11.799	1.587	0.005	0.038	0.008
Fatty acid elongation	38	1	Palmitic acid	11.798	1.587	0.005	0.038	0.008
Fatty acid degradation	39	1	Palmitic acid	11.798	1.587	0.005	0.038	0.008
Biosynthesis of unsaturated fatty acids	36	2	Palmitic acid; 8,11,14-Eicosatrienoic acid	11.789	1.587	0.005	0.038	0.008
Purine metabolism	65	1	Hypoxanthine	9.348	1.587	0.014	0.042	0.018
Synthesis and degradation of ketone bodies	5	1	(R)-3-Hydroxybutyric acid	2.333	1.587	0.228	0.456	0.228
Butanoate metabolism	15	1	(R)-3-Hydroxybutyric acid	2.333	1.587	0.228	0.456	0.228
Hub metabolites								
Biosynthesis of unsaturated fatty acids	36	5	Palmitic acid; Stearic acid;Arachidonic acid; 8,11,14-Eicosatrienoic acid; Docosahexaenoic acid	16.795	1.667	0.001	0.009	0.006
Fatty acid biosynthesis	47	1	Palmitic acid;	15.704	1.667	0.002	0.024	0.006
Fatty acid elongation	38	1	Palmitic acid;	15.704	1.667	0.002	0.024	0.006
Fatty acid degradation	39	1	Palmitic acid;	15.704	1.667	0.002	0.024	0.006
Tryptophan metabolism	41	2	L-Tryptophan; L-Formylkynurenine	13.835	1.667	0.003	0.038	0.010
Mucin type O-glycan biosynthesis	10	1	Uridine diphosphate-N-acetylgalactosamine;	13.130	1.667	0.004	0.045	0.011
Arachidonic acid metabolism	36	2	Arachidonic acid; 8,9-DiHETrE	11.673	1.667	0.007	0.067	0.012
Aminoacyl-tRNA biosynthesis	48	3	L-Leucine; L-Tryptophan; L-Tyrosine	10.032	1.667	0.009	0.079	0.012
Ubiquinone and other terpenoid-quinone biosynthesis	9	1	L-Tyrosine	11.063	1.667	0.009	0.079	0.012
Tyrosine metabolism	42	1	L-Tyrosine	11.063	1.667	0.009	0.079	0.012
Phenylalanine metabolism	10	1	L-Tyrosine	11.063	1.667	0.009	0.079	0.012
Phenylalanine, tyrosine and tryptophan biosynthesis	4	1	L-Tyrosine	11.063	1.667	0.009	0.079	0.012
Riboflavin metabolism	4	1	Riboflavin	9.005	1.667	0.019	0.079	0.023
Valine, leucine and isoleucine degradation	40	1	L-Leucine	7.079	1.667	0.038	0.115	0.041
Valine, leucine and isoleucine biosynthesis	8	1	L-Leucine	7.079	1.667	0.038	0.115	0.041
Steroid hormone biosynthesis	85	1	Estriol	4.407	1.667	0.104	0.115	0.104

FDR, The False Discovery Rate-adjusted *p*-value.

### 3.4. Biomarker analyses: diagnostic and outcome prediction nomogram

**Figure 6:**
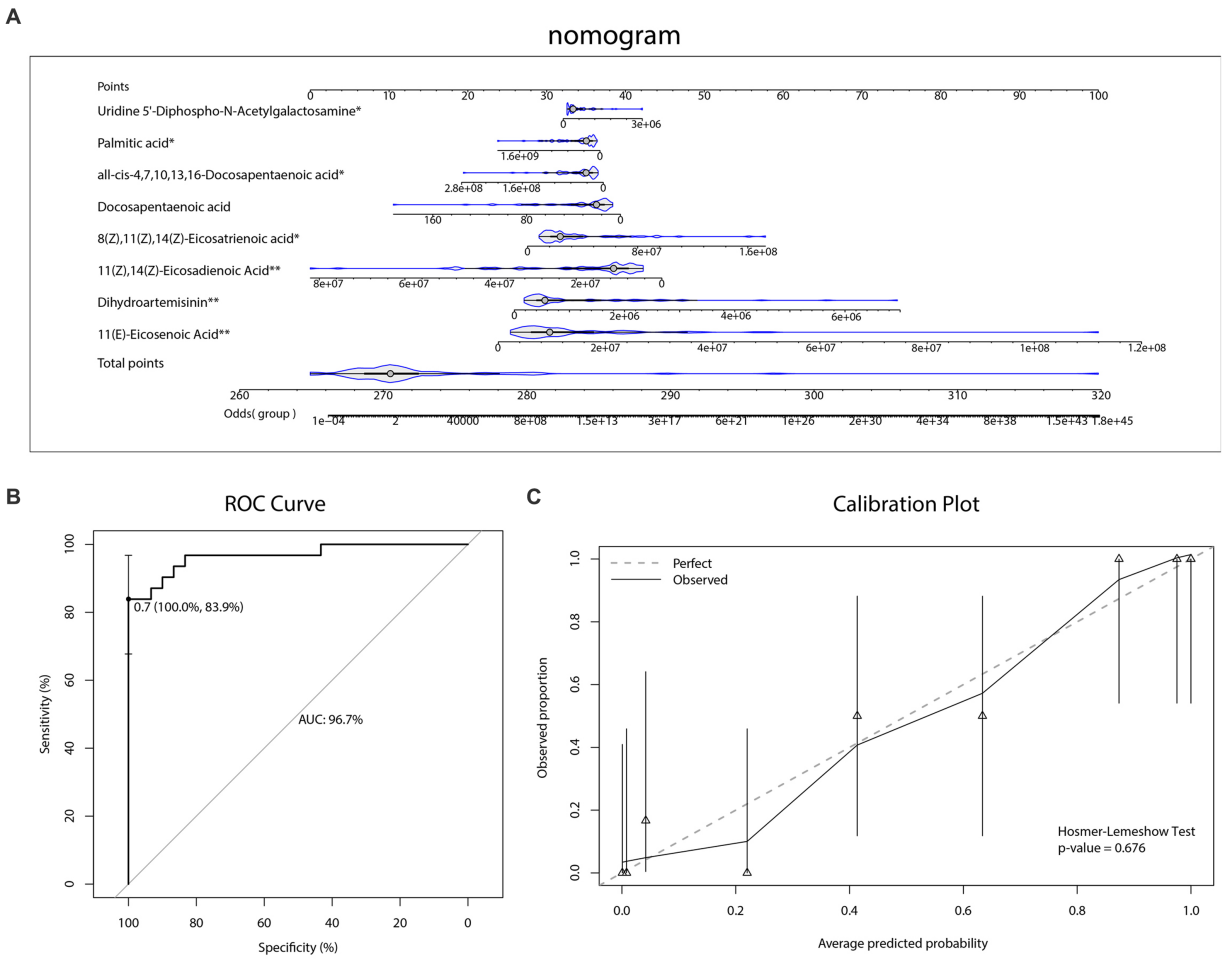
Diagnostic biomarker analysis. **(A)** Diagnostic nomogram. **(B)** The receiver operating characteristic (ROC) curve and the area under the curve (AUC) of diagnostic model. **(C)** The Hosmer–Lemeshow test and calibration plot of diagnostic model.

As Figure 5B and Tables 3, 4 illustrated, there were 13 potential biomarkers which were both differential metabolites and hub metabolites. After stepwise regression, 8 of them were selected as predictor variables which were Uridine 5’-Diphospho-N-Acetylgalactosamine, Palmitic acid, all-cis-4,7,10,13,16-Docosapentaenoic acid, DPA, 8Z,11Z,14Z-Eicosatrienoic acid, 11(Z),14(Z)-Eicosadienoic Acid, Dihydroartemisinin, and 11(E)-Eicosenoic Acid. The nomogram was illustrated in the Figure 6A. The diagnostic performance was evaluated by the ROC curve (Figure 6B), with the AUC value of 96.7% and the 95% confidence interval (CI) ranging from 83.9 to 100%. Besides, the Hosmer–Lemeshow test (*p*-value = 0.676, Figure 6C) showed the goodness of fit for this model.

**Figure 7:**
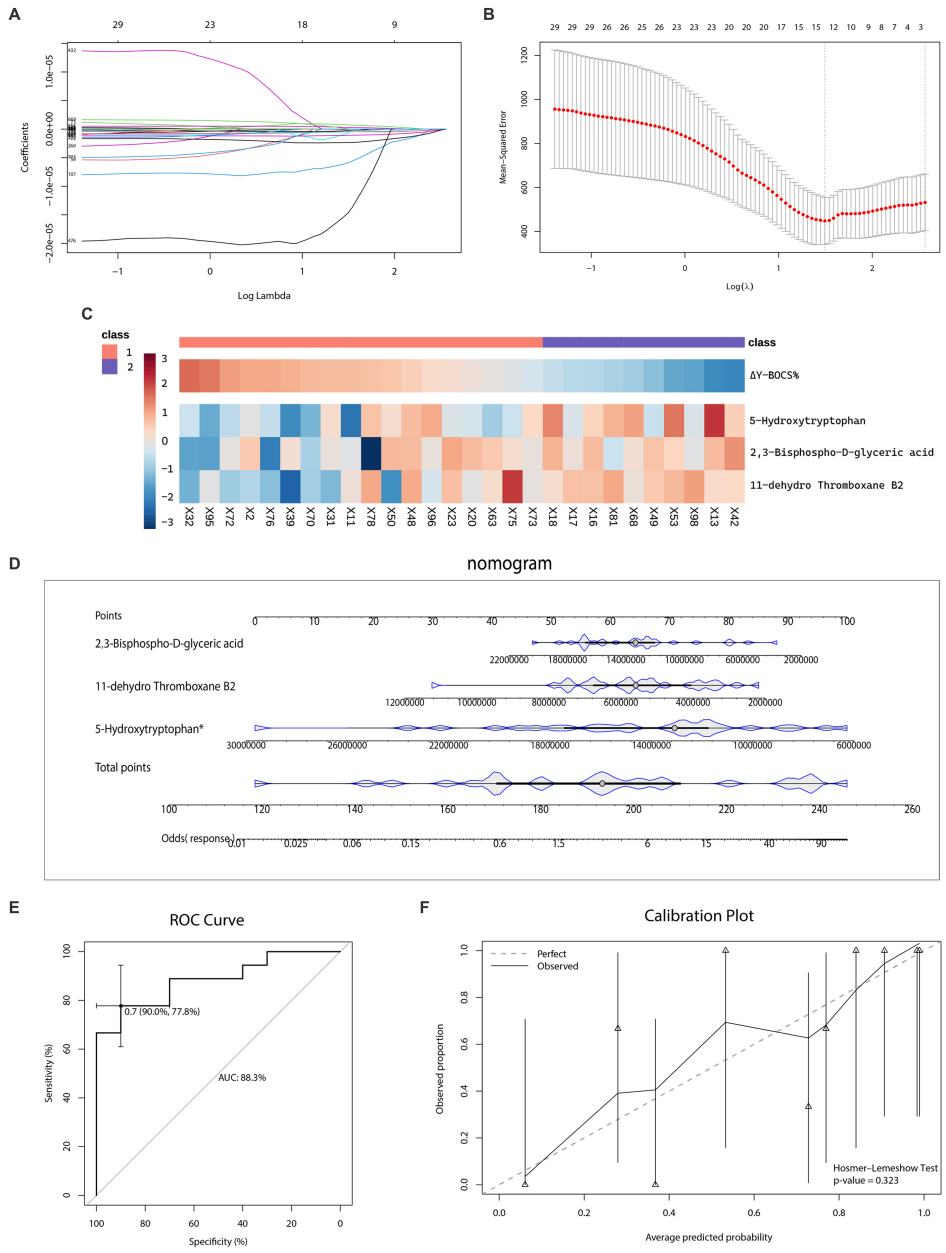
Selection of metabolic predictors and nomogram for predicting the therapeutic effect of sertraline. **(A–B)** Feature selection by the least absolute shrinkage and selection operator (LASSO) regression model. **(C)** The heatmap of metabolic predictors. **(D)** The outcome prediction nomogram. **(E)** The receiver operating characteristic (ROC) curve and the area under the curve (AUC) of outcome prediction model. **(F)** The Hosmer–Lemeshow test and calibration plot of outcome prediction model.

As for the outcome prediction of the sertraline therapeutic effect, a dataset of 28 patients with OCD who received 12-week sertraline monotherapy and finished follow-up visits was utilized for the LASSO analysis (Figures 7A,B). Among a total of 929 recognizable metabolites, three kinds of plasma metabolites were selected, namely 5-Hydroxytryptophan, 11-dehydro Thromboxane B2, 2,3-Bisphospho-D-glyceric acid. The heatmap (Figure 7C) illustrated the level of the selected metabolites in patients with OCD and their reduction rates of Y-BOCS scores.

The nomogram (Figure 7D) visualized the effect of each metabolic predictor. By adding up points of every metabolic predictor, the total points were then calculated and positioned in the bottom line to evaluate the probability of response to sertraline monotherapy in patients with severe OCD. The predictability and consistency of this nomogram were also verified. The accumulated points summarized by the nomogram presented the possibility of the sertraline treatment response. The predictive ability of this model was, evaluated by the AUC value, 88.3% (CI: 77.8–90.0%) (Figure 7E). Meanwhile, we also conducted the Hosmer–Lemeshow test (*p*-value = 0.323), which showed the goodness of fit for this model. The calibration curve was shown in Figure 7F.

## 4. Discussion

To our knowledge, this article was the first application of the untargeted metabolomics approach based on UPLC-Q-TOF/MS technology to analyze the plasma metabolic signature of OCD patients. Our analyses indicated that some amino acids and fatty acids, particularly in the biosynthesis of unsaturated fatty acids and tryptophan metabolism, were significantly different in OCD patients compared with HCs, and may play important roles in the pathogenesis of OCD. Besides, to explore the utility of circulating metabolites as biomarkers, we conducted nomograms of selected metabolites for diagnosis and sertraline treatment prediction based on the logistic regression model and achieved great discriminative abilities.

### 4.1. PUFAs and inflammation in OCD

PUFAs, comprising 25–30% of the fatty acids in the human brain, have been shown to fulfill vital roles in regulating brain inflammation (Janssen and Kiliaan, 2014). Among them, omega-6 PUFAs (n-6) and omega-3 PUFAs (n-3) are two predominant kinds of PUFAs in the brain but play different roles. Omega-6 PUFAs, such as Arachidonic Acid, Docosatrienoic Acid and Eicosatrienoic Acid, produce n-6 eicosanoids (including prostaglandins, leukotrienes, thromboxanes, etc.) which mediate broadly pro-inflammatory effects. Omega-3 PUFAs, such as DHA, Eicosapentaenoic Acid (EPA) and DPA, produce n-3 eicosanoids (including resins and resolvins) which are generally anti-inflammatory (Haag, 2003). Hence, lower levels of omega-6 PUFAs and higher levels of omega-3 PUFAs might have particular importance in maintaining brain inflammation and functioning. Besides, PUFAs cannot be synthesized *de novo* and are mainly supplied by the blood; therefore, levels of circulating PUFAs might reflect on levels of counterparts in the brain and serve as potential biomarkers of several psychiatric diseases (Spector, 2001). Deficiency of circulating omega-3 PUFAs has been found in patients with Major Depression Disorder, Bipolar Disease, Schizophrenia and Alzheimer’s disease and has been seen to be closely associated with mood and cognitive function (Gao et al., 2022).

In the current study, we observed an alteration of unsaturated fatty acids and eicosanoids network (turquoise and red module) among patients with OCD. As shown in the turquoise module, both omega-6 PUFAs (Arachidonic acid, 11(Z),14(Z)-Eicosadienoic Acid, all-cis-4,7,10,13,16-Docosapentaenoic acid, Adrenic acid, 8Z,11Z,14Z-Eicosatrienoic acid, Docosatrienoic Acid) and omega-3 PUFAs (DHA, Docosapentaenoic acid, 11(Z),14(Z),17(Z)-Eicosatrienoic acid) were selected as hub metabolites and tended to be elevated compared to HCs. Whilst, levels of several omega-6 PUFAs (11(Z),14(Z)-Eicosadienoic Acid, all-cis-4,7,10,13,16-Docosapentaenoic acid, Adrenic acid, 8(Z),11(Z),14(Z)-Eicosatrienoic acid) significant increased comparing with HCs. These results suggested that patients with OCD have higher levels of circulating PUFAs but a relatively lower ratio of omega-3 to omega-6, consistent with the inflammatory hypothesis of OCD.

The unbalanced omega-3/omega-6 and pro- and anti- inflammatory processes might be new treatment targets in OCD. According to a randomized double-blind clinical trial, when taking celecoxib as an adjuvant to fluoxetine to inhibit cyclooxygenase-2 (COX-2), an enzyme converts arachidonic acid to eicosanoids, it presents rapid-onset anti–obsession and –compulsion effects in patients with OCD (Sayyah et al., 2011). Whereas, a preliminary placebo-controlled crossover trial adjunctive Omega-3 PUFAs (EPA in this case) failed to bring improvement in obsessive–compulsive, anxiety and depressive symptoms in patients with OCD, unlike its therapeutic benefit in other psychiatry diseases (Janssen and Kiliaan, 2014). What needs to be emphasized is that there were insufficient participants in the two trials mentioned above and more associated studies are called for in the future.

### 4.2. Tryptophan, neurotransmitters and inflammation in OCD

Tryptophan metabolism might be a key driver of the neurobiological mechanism of OCD. As we mentioned before, in the yellow module, Trp, N-Formylkynurenine were screened as hub metabolites and decreased compared with HCs. Trp, the essential amino acid, is a key precursor of serotonin (5-hydroxytryptamine or 5-HT), which is transported into the brain *via* the transporter located in capillaries of the blood–brain barrier and thus different plasma levels of Trp can affect cerebral 5-HT levels (Carneiro et al., 2018). In parallel to existing studies, we found lower but inapparent plasma Trp levels in OCD than HCs (Lucca et al., 1992). Furthermore, acute Trp depletion, which might lead to a short-term increase in presynaptic 5-HT availability, fails to improve the core impulsive-compulsive symptom in a short time (Berney et al., 2006; Külz et al., 2007), but cause a significant decrease perceived control and increase in interfering thoughts at the time of provocation (Hood et al., 2017).

Whereas, only a minor fraction of Trp is utilized for 5-HT synthesis; nearly 95% of the Trp pool enters the kynurenic pathway (Platten et al., 2019). The first stage of the Trp-kynurenic pathway is catalyzed by the hepatic enzyme tryptophan 2,3-dioxygenase (TDO) and the extrahepatic enzyme indoleamine 2,3-dioxygenase (IDO), enzymes that are induced by glucocorticoids and pro-inflammatory cytokines, respectively. Thus, chronic stress and infections can shunt available Trp toward the kynurenic pathway and thereby lower 5-HT synthesis (Sorgdrager et al., 2019), which in combination with the neuroinflammatory hypothesis may contribute to the hyposerotonin status in people with OCD.

Melatonin, which can be biosynthesized by Trp or serotonin and is involved in the control of the sleep–wake cycle, saw significantly lower plasma levels in patients with OCD. Previous studies have also demonstrated a decreased 24-h production of melatonin compared patients with OCD to HCs (Catapano et al., 1992; Millet et al., 1998), contributing to disruptions in circadian rhythms of the sleep–wake cycle. In particular, Delayed bedtimes are associated with more severe OCD symptoms (Coles et al., 2020; Nota et al., 2020).

Notwithstanding, these mentioned metabolites might not be suitable as biomarkers for diagnosis. Melatonin was one of the differential metabolites, but was not a stable predictor variable in our diagnostic nomogram model. Therefore, we proposed that there were wide, subtle and mostly poorly detectable (in separate comparisons) alterations in tryptophan metabolism in the plasma of patients with OCD, but drive an important role through the perturbation of serotonergic neurotransmitters and neuroinflammation in OCD.

### 4.3. Metabolites as promising biomarkers

We performed the logistic regression model using a combination of several selected metabolites to evaluate the discriminative ability of metabolites as promising biomarkers for diagnosis and outcome prediction of sertraline treatment efficacy. The results seemed promising. Both diagnostic and predictive nomogram model had impressive discrimination (AUC value) and calibration (*p*-value from the Hosmer–Lemeshow test) performances.

We discovered, for the first time, the capacity of fatty acids as potential biomarkers for OCD diagnosis. Four out of eight predictor variables of the diagnostic nomogram model are PUFAs. Thereinto, decreased circulating DPA, omega-3 PUFAs, was associated with a lower risk of OCD. Similarly, the protective effects of omega-3 PUFAs have been also found in other psychiatric diseases, such as attention deficit hyperactivity disorder, bipolar disorder, and schizophrenia (Bozzatello et al., 2016).

5-Hydroxytryptophan (5-HTP), meanwhile, was a significant predictor variable of the predictive nomogram model for sertraline treatment. A lower plasma level of 5-HTP was related to higher odds of response after receiving sertraline according to the predictive nomogram model. Also, recent studies revealed the potential benefits of 5-HTP as an augmentative medication of SSRIs (Yousefzadeh et al., 2020). 5-HTP is the intermediate metabolite of the Trp-serotonin pathway and can rapidly be converted to 5-HT. Since 5-HTP passively crosses the blood–brain barrier while 5-HT does not, the low plasma level of 5-HTP might present the hyposerotonin state in the brain which could be reversed by SSRIs.

## 5. Limitations

There were some limitations in our research. Firstly, we only included patients with severe obsessive–compulsive symptoms, because we assumed that the metabolomic profile alterations would be more prominent and typical in patients with severe OCD. But the metabolomic profile of patients with mild to moderate OCD is still unknown; comparisons between patients with severe OCD and mild OCD need to be explored, which might offer fresh and novel insights into the mechanisms of OCD. Secondly, OCD is a heterogeneous disorder with distinct symptom dimensions and the pathological profiles of OCD may differ across symptom dimensions. Dimension-based analysis would be meaningful in future studies with larger sample sizes. As for biomarker analyses, we only validated our diagnostic and predictive model within the given dataset. We lack large-scale, multi-center data and external validation to access models’ reproducibility and generalizability, and therefore, our models should be interpreted with caution. The last but not the least, we did not control the body’s dietary intake, Body Mass Index or other Nutritional Status Indicators during studies.

## 6. Conclusion

Based on untargeted UPLC-Q-TOF/MS analysis, we discovered unsaturated fatty acids and tryptophan metabolism as key metabolic pathways alteration in OCD, and circulating metabolites of these pathways, such as DPA and 5-HTP, appeared to be promising biomarkers for OCD identification and sertraline treatment prediction.

## Data availability statement

The raw data supporting the conclusions of this article will be made available by the authors, without undue reservation.

## Ethics statement

The studies involving human participants were reviewed and approved by The Institutional Review Boards of Shanghai Mental Health Center. The patients/participants provided their written informed consent to participate in this study.

## Author contributions

ZL and ZW were involved in the study design and writing of the manuscript. JG, LL, ZZ, and SY contributed to the acquisition of data for the work. ZL, DS, WW, and ZW were involved in the analysis and interpretation of the results. All authors contributed to the article and approved the submitted version.

## Funding

This study was funded by the Shanghai Municipal Education Commission-Gao Feng Clinical Medicine Grant (20161321), the grants from Shanghai Municipal Health Commission (2019ZB0201), and the Shanghai Key Laboratory of Psychotic Disorders (No.13dz2260500).

## Conflict of interest

The authors declare that the research was conducted in the absence of any commercial or financial relationships that could be construed as a potential conflict of interest.

## Publisher’s note

All claims expressed in this article are solely those of the authors and do not necessarily represent those of their affiliated organizations, or those of the publisher, the editors and the reviewers. Any product that may be evaluated in this article, or claim that may be made by its manufacturer, is not guaranteed or endorsed by the publisher.

## Footnotes

^1^ https://www.metaboanalyst.ca/

^2^ https://www.mzcloud.org/
