# Supplementary material for: Untargeted metabolomics analysis in drug-naïve patients with severe obsessive–compulsive disorder
Source: Front Neurosci. 2023 Jun 2;17:1148971. doi: 10.3389/fnins.2023.1148971 (PMC10272357; doi:10.3389/fnins.2023.1148971)
Supplement: Supplementary file 1 [file Table_1.DOCX]

Supplementary Material

Untargeted metabolomics analysis in drug-naïve patients with severe obsessive-compulsive disorder

Zheqin Li^1^, Jian Gao^1^, Liangjun Lin^1^, Zifeng Zheng^1^, Susu Yan^2^, Weidi Wang^3,4,5^, Dongdong Shi^1,4^, Zhen Wang^1,4,6*^

^1^Shanghai Mental Health Center, Shanghai Jiao Tong University School of Medicine, Shanghai, China;

^2^Shandong Daizhuang Hospital, Jining, Shandong, China

^3^Shanghai Mental Health Center, School of Biomedical Engineering, Shanghai Jiao Tong University School of Medicine, Shanghai, China.

^4^Shanghai Key Laboratory of Psychotic Disorders, Shanghai Mental Health Center, Shanghai, China.

^5^Brain Science and Technology Research Center, Shanghai Jiao Tong University, Shanghai, China.

^6^Institute of Psychological and Behavioral Science, Shanghai Jiao Tong University, Shanghai, China

*** Correspondence** Zhen Wang: wangzhen@smhc.org.cn

# Supplementary Figures

**Supplementary Figure 1.** Outliers detection in data of positive and negative polarity modes. **(A)**The principal component analysis of metabolites in positive polarity modes. **(B)** The heatmap of metabolites in positive polarity modes. **(C)** The principal component analysis of metabolites in negative polarity modes. **(D)** The heatmap of metabolites in negative polarity modes.
