# Supplementary figures and images for: Untargeted metabolomics analysis in drug-naïve patients with severe obsessive–compulsive disorder
Source: Front Neurosci. 2023 Jun 2;17:1148971. doi: 10.3389/fnins.2023.1148971 (PMC10272357; doi:10.3389/fnins.2023.1148971)

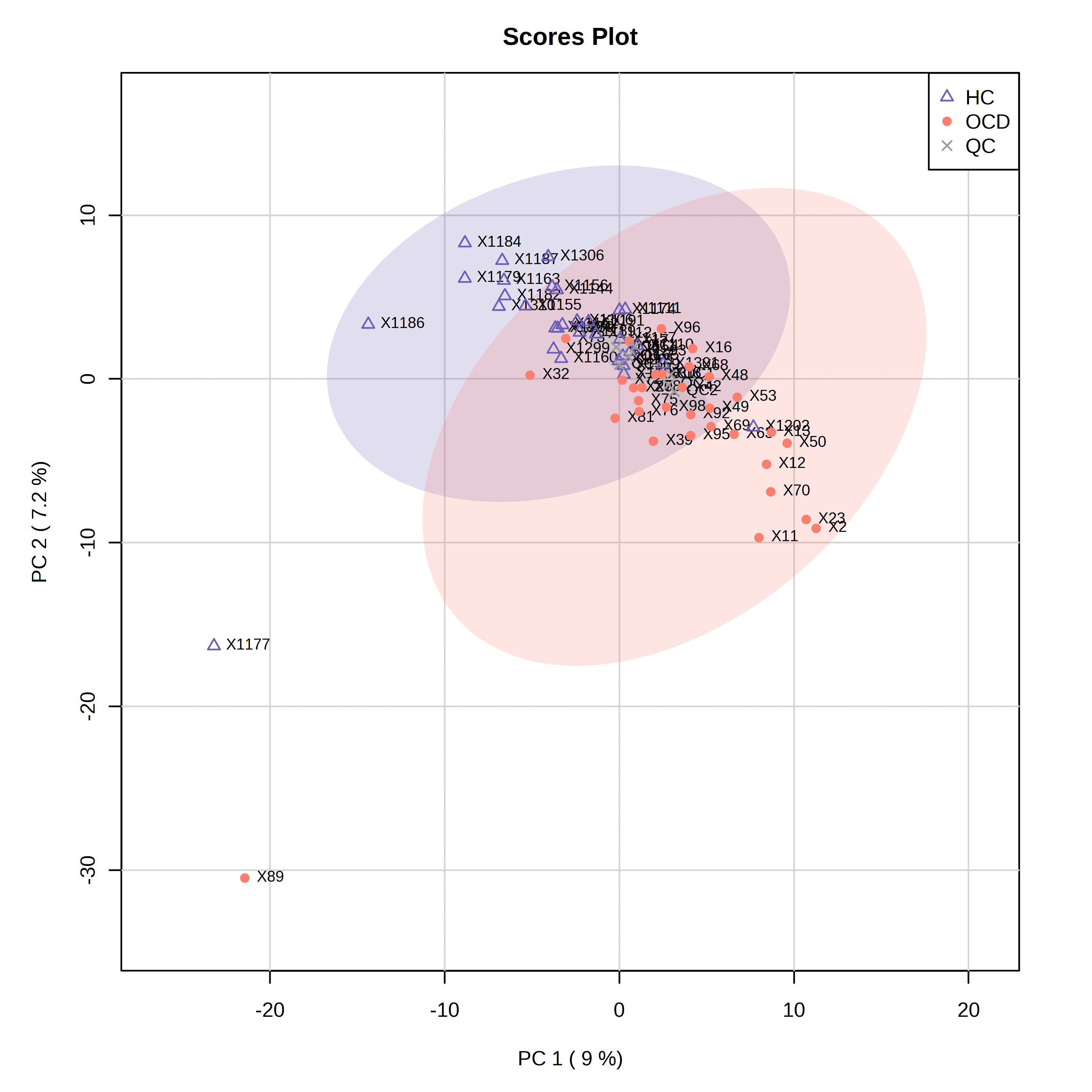

Supplement: Supplementary file 2 [file Data_Sheet_1.ZIP › Supplementary Figures/sFIGURE1A.png]

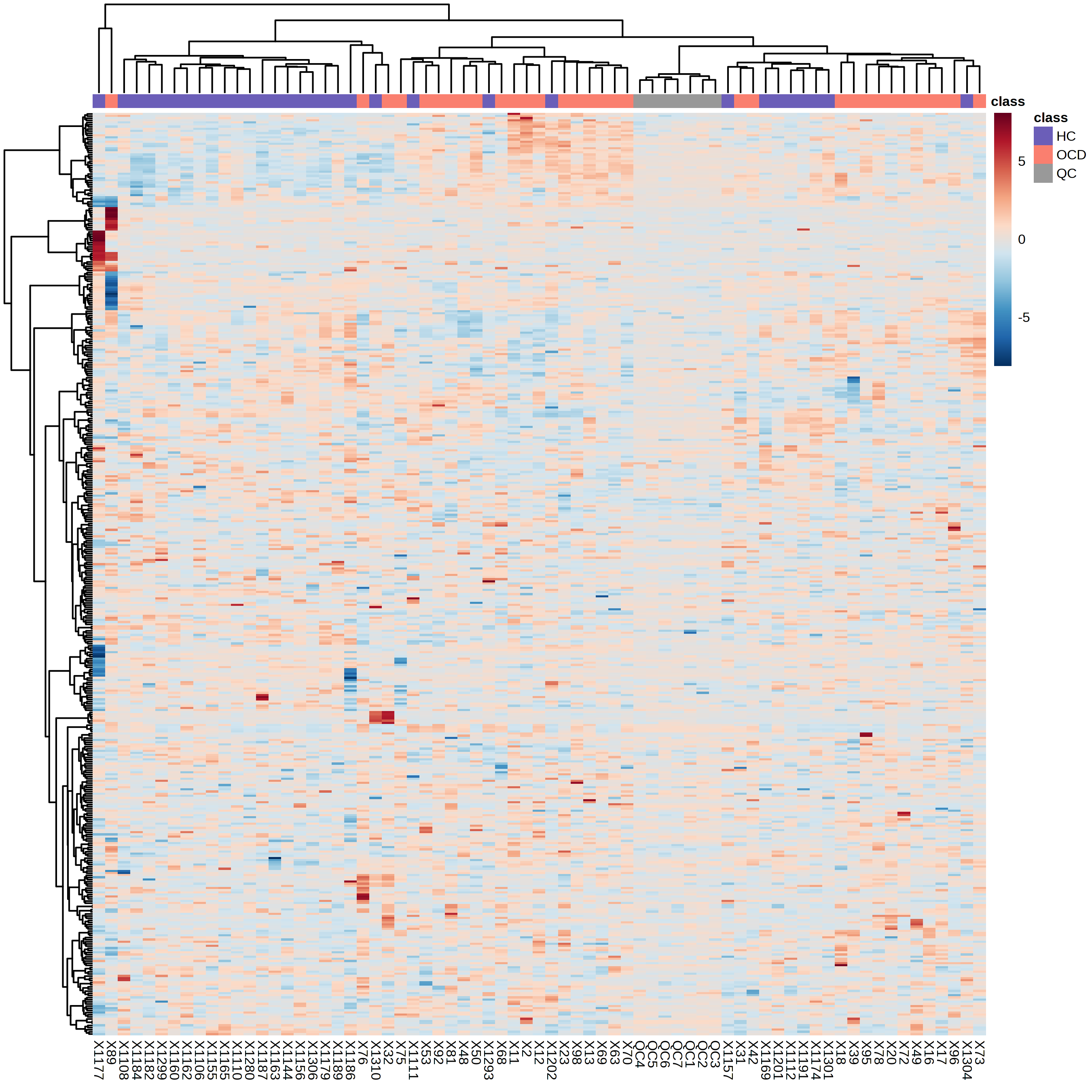

Supplement: Supplementary file 2 [file Data_Sheet_1.ZIP › Supplementary Figures/sFIGURE1B.png]

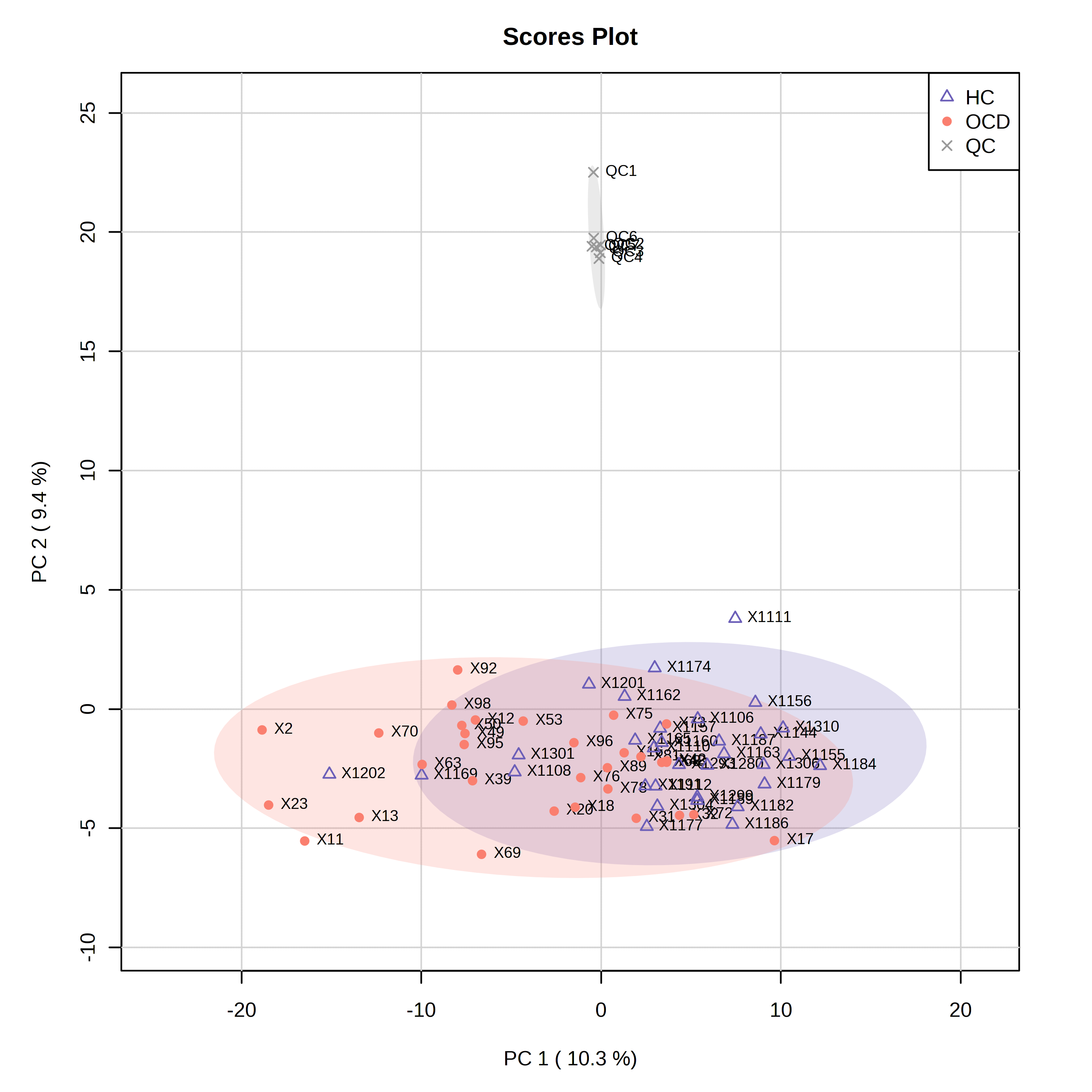

Supplement: Supplementary file 2 [file Data_Sheet_1.ZIP › Supplementary Figures/sFIGURE1C.png]

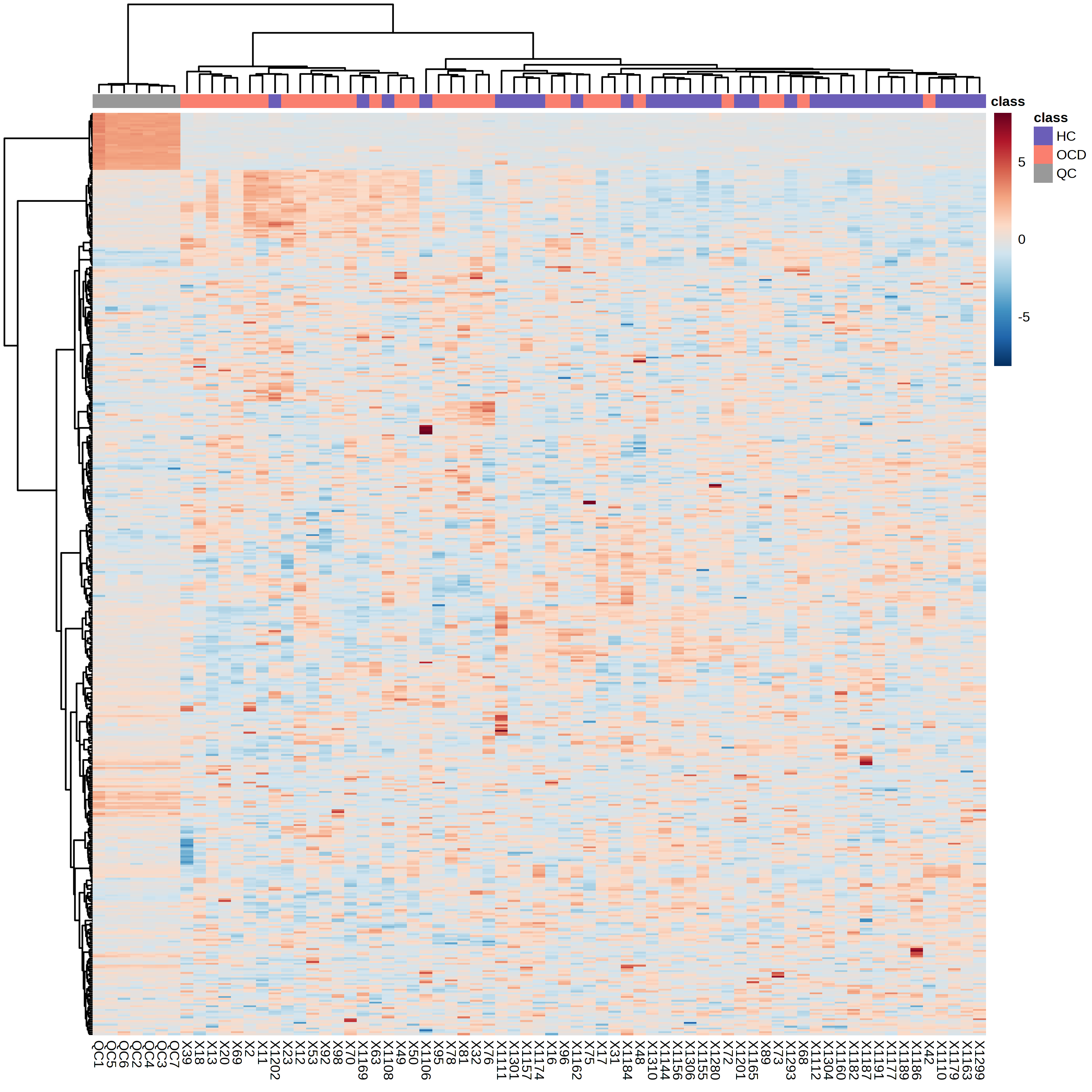

Supplement: Supplementary file 2 [file Data_Sheet_1.ZIP › Supplementary Figures/sFIGURE1D.png]
